# Management of Takotsubo Syndrome: A Comprehensive Review

**DOI:** 10.7759/cureus.6556

**Published:** 2020-01-03

**Authors:** Yasar Sattar, Kelvin Shenq Woei Siew, Michael Connerney, Waqas Ullah, M Chadi Alraies

**Affiliations:** 1 Internal Medicine, Icahn School of Medicine at Mount Sinai, New York, USA; 2 Cardiology, University of Malaya, Kuala Lumpur, MYS; 3 Internal Medicine, Abington Hospital - Jefferson Health, Abington, USA; 4 Cardiology, Detroit Medical Center, Detroit, USA

**Keywords:** takotsubo cardiomyopathy, takotsubo syndrome, cardiomyopathy, left ventricular outflow tract obstruction, acute coronary syndrome

## Abstract

Takotsubo syndrome (TTS), also known as Takotsubo cardiomyopathy, is a transient left ventricular wall dysfunction that is often triggered by physical or emotional stressors. Although TTS is a rare disease with a prevalence of only 0.5% to 0.9% in the general population, it is often misdiagnosed as acute coronary syndrome. A diagnosis of TTS can be made using Mayo diagnostic criteria. The initial management of TTS includes dual antiplatelet therapy, anticoagulants, beta-blockers, angiotensin-converting enzyme inhibitors or aldosterone receptor blockers, and statins. Treatment is usually provided for up to three months and has a good safety profile. For TTS with complications such as cardiogenic shock, management depends on left ventricular outflow tract obstruction (LVOTO). In patients without LVOTO, inotropic agents can be used to maintain pressure, while inotropic agents are contraindicated in patients with LVOTO. In TTS with thromboembolism, heparin should be started, and patients should be bridged to warfarin for up to three months to prevent systemic emboli. Our comprehensive review discussed the management in detail, derived from the most recent literature from observational studies, systematic review, and meta-analyses.

## Introduction and background

Takotsubo syndrome (TTS), also commonly known as Takotsubo cardiomyopathy or apical ballooning syndrome, is characterized by transient left ventricular (LV) wall dysfunction triggered by physical or emotional factors with similar presentation features as acute coronary syndrome (ACS) [1]. TTS accounts for 0.02% of the inpatient admission in the United States [2]. The prevalence of TTS in the general population, mimicking ST-segment elevation myocardial infarction (STEMI), is estimated to be 0.5% to 0.9%, with a higher prevalence in women in their sixth decade of life. Indeed, 2% to 3% of STEMI presentations in women turn out to be TTS every year [2-4].

As the clinical presentation of TTS is often indistinguishable from ACS, it is a diagnosis of exclusion once true ACS is ruled out with coronary angiography. According to Luscher and Templin et al., TTS and ACS commonly present with chest pain and dyspnea on exertion [1,5]. However, most cases of TTS have an identifiable risk factor of physical or emotional stress that can differentiate it from ACS. To date, there is no noninvasive test that can confirm the diagnosis of TTS. The definitive diagnostic tool to differentiate TTS from ACS is coronary angiogram. Furthermore, an LV angiogram has classic features which include apical rounding, followed by basal and mid-ventricular contraction. Upon establishing the diagnosis, continuous telemetry monitoring and repeated echocardiograms are essential to identify complications of TTS. Some of the complications of severe TTS that can affect ionotropic function include acute heart failure, LV outflow tract obstruction (LVOTO), cardiogenic shock, and the presence of LV thrombus. Other complications of TTS can include rhythm abnormalities such as atrial fibrillation, ventricular fibrillation, atrioventricular block, and cardiac arrest [5]. Our review discusses current management approaches for TTS during the acute and long-term phases of the disease.

## Review

Literature search and data source

A systematic search was performed on PubMed, MEDLINE, Cochrane Library, and Google Scholar databases. The search included MeSH terms for ‘Takotsubo Cardiomyopathy’, ‘Takotsubo Syndrome’, ‘Broken Heart Syndrome’, ‘Stress Cardiomyopathy’, ‘Transient Apical Ballooning Syndrome’, ‘Apical Ballooning Syndrome’, ‘'Left Ventricular Apical Ballooning Syndrome’, ‘Treatment’, and ‘Management’. The MeSH terms were combined using Boolean operators AND and OR. The studies included were from inception to July 31, 2019. Inclusion criteria were studies that are in English language or foreign languages that are translated into English, and articles that are focused on the management of TTS with measurable outcomes. Exclusion criteria were single case reports and management with no measurable outcomes or follow-up. Two physicians conducted a literature search and independently screened all titles and abstracts. A total of 4,313 articles were found of which 248 articles met the inclusion criteria. Based on predefined criteria, only 16 of these articles were included in this review (Table 1) [5-20].

**Table 1:** Studies included in this review regarding clinical outcomes of management of Takotsubo syndrome ACE, angiotensin-converting enzyme (inhibitors); ALA, alpha-lipoic acid; ARB, aldosterone receptor blocker; CR, cardiac rupture; CRP, C-reactive protein; LMWH, low molecular weight heparin; LV, left ventricular; LVEF, left ventricular ejection fraction; LVOT, left ventricular outflow tract; LVOTO, left ventricular outflow tract obstruction; MACE, major adverse cardiac event; MIBG, metaiodobenzylguanidine; MRA, mineralocorticoid receptor antagonists; N/A/, not applicable; OAC, oral anticoagulants; OD, once daily; TIA, transient ischemic attack; TNF, tumor necrosis factor; TTS, Takotsubo syndrome [5-20]

Authors, Year of Publication	Study Design: Number of Subjects (n), Area of Study	Treatment Regimen	Primary Measure Studied and Follow-Up Duration	Study Outcomes and Conclusions
Templin et al. 2015	Retrospective observational study: n=1,750 (all TTS)	Beta-blockers and ACE	Outcome measure: MACE. Follow-up: 30 days and 10 years	Study outcome: ACE inhibitor or ARB improve one-year survival. Conclusion: ACE or ARB is beneficial, beta-blockers not beneficial
Santoro et al. 2017	Prospective cohort study: n=12, TTS with LV thrombi	Acute phase (in hospital): LMWH followed by enoxaparin. Long term: OAC (warfarin) for 3 months, discontinuation upon resolution of TTS	Outcome measures: Acute: cerebrovascular embolic event. Long term: new event of stroke, overall survival. Follow-up: 984 days	Study outcomes: Acute phase: LMWH beneficial in stroke prevention. Long term: interruption of OAC after three months with no new stroke, similar survival with or without LV thrombi. Conclusions: LMWH beneficial in acute phase, OAC reasonable use as stroke prophylaxis up to three months
Marfella et al. 2016	Randomized controlled trial: n=48 (all TTS)	ALA 600 mg OD vs placebo	Outcome measures: quantitative MIBG imaging for adrenergic cardiac innervation improvement; Reduction in inflammation marker (CRP, TNF, nitrotyrosine level). Follow-up: one year	Study outcomes: MIBG imaging defect size reduction was greater in ALA treated group compared to placebo; ALA treated group had reduction in inflammation marker compared to placebo. Conclusion: ALA is beneficial
Yeyehd et al. 2016	Observational study: n=117 (all TTS)	Acute phase: Aspirin, Clopidogrel, Fondaparinux, Statin, beta-blockers, ACE/ARB. Discharge: ACE/ARB, beta-blockers, aspirin, clopidogrel, statin + psychological management	Outcome measures: in-hospital mortality; one-year hospital readmission; recurrence of TTS. Follow-up: one year	Study outcomes: no in-hospital mortality; 2.8% re-hospitalization with heart failure; no recurrence of TTS. Conclusion: the standard regime is beneficial, consider discontinued antiplatelet at discharge if TTS diagnosis is certain
Ansari et al. 2018	Observational study: n=114 TTS with hemodynamic instability	With or without catecholamine support in-hospital	Outcome measures: in-hospital mortality; long-term mortality. Follow-up: Four years	Study outcomes: patients require catecholamine support higher in-hospital and long-term mortality; higher 30 day and long-term mortality. Conclusion: catecholamine use for circulatory support possibly exacerbates the risk of mortality
Santoro et al. 2016	Case-controlled study: n=9 TTS with LVOTO	IV esmolol infusion 0.15 0.3 mg/ kg/ min for 24 hours after admission, bisoprolol 1.5 mg daily. Case-controlled study: n=9 TTS with LVOTO	Outcome measures: LVOT pressure gradient; systolic blood pressure. Follow-up: nine months	Study outcomes: esmolol infusion associated with reduction LVOT gradient and systolic blood pressure. Conclusion: esmolol infusion and bisoprolol is possibly beneficial in TTS with LVOTO
Abanador-Kamper et al. 2017	Observational study: n=72 (all TTS)	Different combination antithrombotic therapy (aspirin, P2Y12 antagonist, OAC and LMWH) for 3,6 or 12 months) + Heart failure regimen (ACE, beta-blocker, MRA) at discharge	Outcome measures – MACE: in-hospital/ Long-term mortality, stroke, myocardial infarction, recurrent TTS. Follow-up: 24 months	Study outcomes: moderate MACE, an event rate of 12%, 1% in-hospital mortality, 5% two-year all-cause mortality. Conclusion: beneficial antithrombotic therapy + heart failure regime for at least two months
Isogai et al. 2016	Observational study: n=2,672 (all TTS)	Early beta-blockers use who started on day one or two of hospitalization compared to no beta-blocker treatment during hospitalization (control group)	Outcome measure: 30-day in-hospital mortality. Follow-up: in-hospital until 30-day after admission	Study outcomes: no mortality benefit for early beta-blocker using compared to control group. Conclusion: early beta-blocker not beneficial
Francesco et al. 2014	Meta-analysis: n=8 studies (all TTS studies with a median follow up of three years)	Standard pharmacological therapy (beta-blockers, ACE/ARB, aspirin. and statins)	Outcome measures: recurrence of TTS at follow up. Follow-up: median three years	Study outcomes: All four pharmacological therapies do not significantly reduce recurrence of TTS. Conclusion: beta-blockers, ACE, ARB, aspirin, and statins are not beneficial in reducing recurrence of TTS
Kumar et al. 2011	Systematic review: n=11 case reports of TTS with CR	Use of beta-blockers of patient with cardiac rupture compared to control group	Outcome measures: N/A. Follow up: N/A	Study outcomes: TTS who developed CR associated with lower use of beta-blockers compared to control group (mean: 36% vs 86%), P = .03. Conclusion: beta-blocker use may have protective effect against CR and may be useful in TTS patients
Regnante et al. 2009	Observational study: n=70 (all TTS)	Standard cardiovascular medication (aspirin, beta-blockers, ACE, statin). Discharged with warfarin for TTS with severe apical wall motion abnormalities	Outcome measures: MACE; recurrence of TTS. Follow-up: four years	Study outcomes: long-term use of ACE before TTS onset protective against cardiogenic shock, sustained ventricular arrhythmia and death; beta-blockers not protective against recurrence of TTS. Conclusion: long-term use of ACE may be beneficial/protective against TTS, beta-blockers not beneficial against recurrent TTS
Fazzio et al. 2008	Observational study: n=33 (all TTS)	Beta-blockers, ACE inhibitors, aspirin, or calcium channel blockers compared to control	Outcome measures: LVEF functional improvement; days of hospitalization. Follow-up: 30 days	Study outcomes: no significant difference found between treatment group and control group. Conclusion: All four medications are not beneficial
de Gregorio. 2010	Systematic review: n=36 TTS with LV thrombus	Anticoagulation	Outcome measures: any cardioembolic event (stroke, TIA, renal infarct, peripheral ischemia). Follow-up: N/A	Study outcome: Early anticoagulation treatment with suspected TTS at risk of thromboembolic diseases, irrespective for presence of LV clot. Conclusion: anticoagulation is beneficial in TTS with risk of thromboembolism
Santoro et al. 2013	Case series: n=13 (all TTS)	IV levosimendan 0.1 mcg/kg/min	Outcome measures: LVEF; any adverse event. Follow-up: 441 days	Study outcome: all had improved LVEF on third day and discharge compared to admission; 15% had adverse event. Conclusion: levosimendan possibly beneficial in improving LVEF
Dias et al. 2016	Retrospective study: n=206 (all TTS)	Antiplatelet (single/dual), beta-blockers, ACE, or statin	Outcome measure: MACE (in-hospital heart failure, death, stroke or respiratory failure). Follow-up: until discharge	Study outcome: single or dual antiplatelet therapy independent predictors of lower incidence of MACE. Conclusion: antiplatelet therapy beneficial
Singh et al. 2014	Systematic review and meta-analysis: n=847 (all TTS)	Beta-blockers and ACE	Outcome measure: recurrence rate. Follow-up: N/A	Study outcome: TTS recurrence inversely correlated with ACE prescription and independent of beta-blockers. Conclusion: ACE beneficial

**Figure 1:**
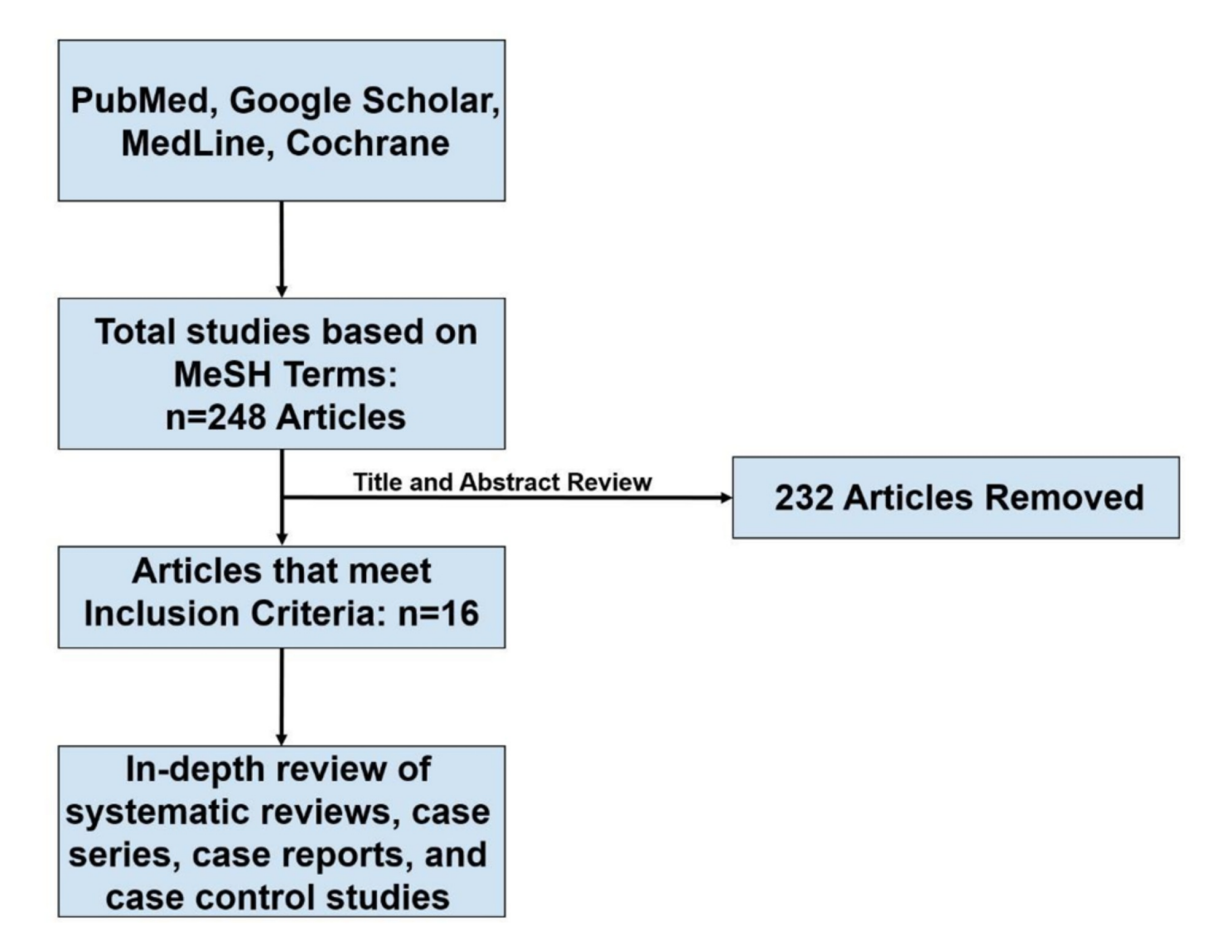
The PRISMA search strategy of the review PRISMA, Preferred Reporting Items for Systematic Reviews and Meta-Analyses

A detailed search breakdown is shown in a Preferred Reporting Items for Systematic Reviews and Meta-Analyses (PRISMA) flow diagram (Figure 1).

Our review of TTS is divided into a short discussion about diagnosis and an extended discussion about management. The extended discussion is divided into TTS with and without complications.

Diagnosis of TTS

The diagnosis of TTS should be based on differentials in patients with ACS symptoms such as chest pain or dyspnea on exertion with positive cardiac biomarkers, including troponin, particularly when echocardiographic and clinical manifestations do not fit the pattern of ACS [21]. A careful history review of physical or emotional stressors should be taken. However, TTS can present without any inciting emotional or physical stressors. A diagnosis of TTS is met if the patient meets all four Mayo Clinic criteria; these can be helpful in its diagnosis (Table 2) [22-23].

**Table 2:** Mayo diagnostic criteria LV, left ventricular; EKG, electrocardiogram [22-23]

	Mayo Criteria
1	Transient regional LV wall dysfunction (dyskinesia, hypokinesia, and akinesia) with deficits extending beyond a single epicardial contribution; with a rare exception of focal and global type
2	New ST elevation or T-wave inversion on EKG or troponin elevation
3	Absence of angiographic evidence of plaque or coronary obstruction
4	Absence of myocarditis or pheochromocytoma

A diagnosis of TTS should also be considered if wall motion defects do not correlate with an obstructed coronary artery. The exception is a percentage of patients that have concurrent obstructed coronary artery disease (CAD) and TTS; per the International Takotsubo Registry, this subset included 15.3% of TTS cases [5]. Thus, a diagnosis of TTS that includes an electrocardiogram and troponin results can resemble ACS.

Diagnostic Imaging

**Figure 2:**
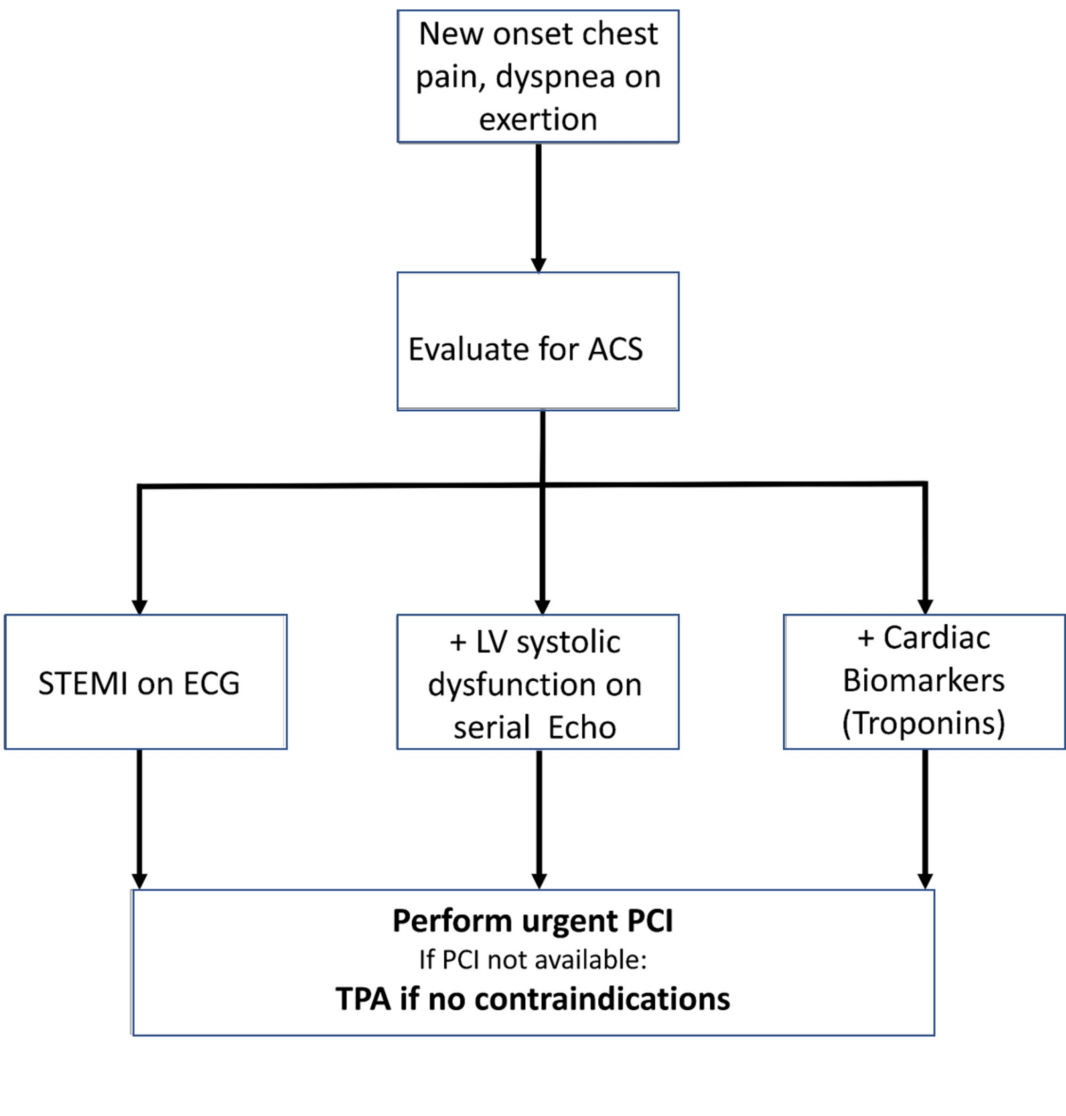
TTS diagnosis during the evaluation of ACS ACS, acute coronary syndrome; LV, left ventricular; PCI, percutaneous coronary intervention; STEMI, ST-segment elevation myocardial infarction; TPA, tissue plasminogen activator; TTS, Takotsubo syndrome

Coronary angiogram is useful for diagnosing TTS. Serial LV systolic function assessment can be done with serial echocardiograms, a one-time assessment with coronary ventriculography, and then cardiac magnetic resonance imaging. It is imperative to suspect TTS during the evaluation of ACS. The approach detailed in Figure 2 should be considered for TTS diagnosis during the evaluation of ACS.

Detection of wall defects on echocardiography or LV ventriculography suggests a review of the following wall defect patterns (Table 3) [5,24-26].

**Table 3:** Wall motion abnormalities in TTS LV, left ventricular; TTS, Takotsubo syndrome [5], [24-26]

Location	Wall motion abnormality
Apical	The most common type of pattern of wall defect found in the TTS International Takotsubo Registry. There is significant LV systolic apical ballooning with mid/apical hypokinesia and often basal hyperkinesia.
Mid-ventricular	The second most common pattern of wall defect found in the International Takotsubo Registry. There is LV hypokinesia/wall defect only in LV mid-ventricular region with apical sparing.
Basal	The third most common type of pattern of wall defect found in the International Takotsubo Registry. There is basal hypokinesia of LV with sparing of mid-ventricular and apical region.
Focal	The fourth most common type of pattern of wall defect found in International Takotsubo Registry. In this type, isolated anterolateral segment dysfunction of LV is found.
Global	In rare circumstances, TTS patients can have global hypokinesia.

Cardiac Magnetic Resonance

Cardiac magnetic resonance can highlight the absence of late gadolinium enhancement in TTS as compared to the presence of late gadolinium enhancement seen in transmural or subendocardial regions in myocardial infarction [25].

Management

General Management

The management of TTS is generally conservative and focused on emotional or physical stress relief. However, in some cases, acute complications such as heart failure and shock develop, and intensive management is required. The management of heart failure and shock is similar to that in the general guidelines of their management in non-TTS cases except in the presence of LVOTO. In cases of LVOTO, caution should be taken in preventing volume depletion or with vasodilatory medications. Generally, the duration of treatment of TTS ranges from three months to one year depending on expert opinion. In a study by Fazio et al., it was found that TTS treatment of more than three months did not add any significant change to survival and should be avoided [16].

The management of TTS is divided into TTS without complications and TTS with complications. These complications include hypotension and cardiogenic shock with heart failure or LVOTO, cardiac arrhythmias, and thromboembolism.

TTS without Complications

After a comprehensive assessment, if patients are hemodynamically stable with no other signs of complications such as acute heart failure, arrhythmia, or thromboembolism, they should be admitted to cardiology for further monitoring. In a study by Yeyehd et al., the acute and post-discharge management of TTS with dual antiplatelet therapy (DAPT) including aspirin and clopidogrel, anticoagulation such as low molecular weight heparin or fondaparinux, beta-blockers, statins, angiotensin-converting enzyme (ACE) inhibitors/angiotensin receptor blockers (ARBs), and psychological stress relief management helped to lower inpatient mortality and recurrence of TTS [8]. Furthermore, a study by Yeyehd et al. indicated that DAPT should be discontinued at discharge if TTS is certain [8]. A further review also revealed a study by Dias et al. indicating that single antiplatelet therapy or DAPT are independently helpful in lowering major adverse cardiovascular events/complications in TTS patients [19]. In a study by Templin et al. ACE/ARBs were associated with improved survival at one-year follow-up in patients with and without heart failure in TTS [5]. A study by Regnante et al. showed that the use of ACE inhibitors before the onset of TTS prevented cardiogenic shock, arrhythmias, and death [15]. A meta-analysis by Santoro et al. reviewed three-year follow-up data and concluded that aspirin, statin, beta-blocker, and ACE/ARBs do not reduce the recurrence of TTS [13].

TTS with Complications - Hypotension and Cardiogenic Shock

For TTS patients with hypotension or shock, continuous monitoring of clinical parameters such as vital signs, mental status, urine output, and renal function should be done along with telemonitoring. Up to 5%-10% of patients with TTS can develop cardiogenic shock [5,27]. A variety of factors are associated with cardiogenic shock in TTS, including physical triggers, young age, and low LV ejection fraction. Mortality is higher in TTS with shock by roughly 10-fold [28,29]. A clinical discordance is found between shock and systolic function of the left or right ventricle. This discordance can be due to the presence of LVOTO [30-33]. An urgent echocardiogram should be done in TTS with cardiogenic shock to look for LVOTO and to rule out the systolic anterior motion of the mitral valve or mitral regurgitation [31,33].

The management of hypotension or cardiogenic shock can be further divided into cases with and without LVOTO as follows.

Cardiogenic shock without LVOTO: In patients with hypotension without pulmonary congestion, cautious fluid resuscitation can be performed, even in patients with or without LVOTO. In patients with LV systolic dysfunction but without LVOTO, a trial of inotropic agents such as dopamine or dobutamine can serve as a temporary measure; however, an inotropic agent can cause mild LVOTO in these patients [34]. In moderate to severe LVOTO, pressor/inotropic agents are contraindicated. Patients with persistent hypotension or any signs of end-organ damage should be closely monitoring with pulmonary catheterization/wedge pressure and intra-aortic balloon pumps (IABP). This subset of patients requires vasopressors.

Cardiogenic shock with LVOTO: In patients with cardiogenic shock with moderate to severe LVOTO, inotropic agents should not be used because they can increase the degree of obstruction [31,34]. In a study by Ansari et al., it was reported that inotropes such as epinephrine, norepinephrine, dobutamine, and dopamine should be avoided in TTS, and that catecholamine excess can increase 30-day and long-term mortality in TTS [9]. Only Marfella et al. reported that alpha-adrenergic stimulation by alpha-lipoic therapy could increase sympathetic stimulation of the heart and show improvement of cardiac defects on 123I-metaiodobenzylguanidine myocardial scintigraphy at a 12-month follow-up in TTS patients [7].

The recommended treatment of TTS with moderate to severe LVOTO is similar to hypertrophic cardiomyopathy. The management of this subset of patients includes fluid resuscitation in the absence of pulmonary congestion [22,31]. The use of beta-blockers also plays a role in the relief of obstruction. A case-control study by Santoro et al. suggested that intravenous administration of esmolol at admission, followed by daily bisoprolol can improve LVOTO gradient and relieve obstruction [10].

In TTS with severe LVOTO, alpha agonists such as phenylephrine can help in a closely monitored setting by increasing afterload. However, phenylephrine should be used with caution as it can cause coronary vasospasm. Patients with severe/persistent hypotension not responsive to fluid resuscitation or initial measure with an intra-aortic balloon pump should be closely monitored. As IABPs can decrease afterload, this effect should be monitored closely [32,34]. A further literature review showed several case reports that demonstrated the efficacy of extracorporeal membrane oxygenation as a life-saving alternative treatment for circulatory support in TTS patients with cardiogenic shock [35-37].

TTS with Complications - Heart Failure

Acute heart failure (HF) in TTS is managed the same way as HF from any other illness: by oxygen, respiratory support as needed, and preload and afterload reduction. The exception to the regular management is that preload and afterload reduction therapies should be avoided in cases with LVOTO. In TTS patients with HF without LVOTO, the standard cocktail of medications for HF can be prescribed. This includes diuretics, ACE inhibitors, or ARBs [22]. Beta-blockers do not lower mortality in HF due to TTS. In an observational study of 2,672 patients by Isogai et al., beta-blockers were unable to lower 30-day in-patient mortality in 423 patients [12]. A beneficial effect of beta-blockers is a low incidence of cardiac rupture in TTS treated with beta-blocker, as reported by Kumar et al. [14]. A meta-analysis by Singh et al. concluded that ACE inhibitors prevent the recurrence of TTS, while beta-blockers do not [20].

The recommended duration of treatment is not well known, but in general, treatment with HF medication is four weeks until systolic function improves. A Mayo clinic study failed to show any survival benefits of ACE inhibitors and beta-blockers in TTS patients [38]. An observation study by Abanador-Kamper et al. reviewed all the treatment regimens for TTS patients and concluded that the benefits of heart failure cocktail medications and antithrombotic therapy is highest during the first two months of TTS, with a low side effect profile, high survival, and early recovery [11]. A case series study by Santoro et al. concluded that the use of levosimendan can improve the LV ejection fraction and has a good safety profile [18].

TTS with Complications - Thromboembolism

Ventricular thrombus is found in 1.3% of the 1,750 TTS patients in the International Takotsubo Registry [5]. The risk of LV thrombus should be evaluated by echocardiography in TTS patients with severe LV dysfunction. There are no clear guidelines for anticoagulation to prevent LV thrombus, but some reports have shown that 10 days of anticoagulation can reduce the incidence of LV thrombus in TTS patients. In patients with severe LV dysfunction and/or with LV thrombus, anticoagulation for three months until resolution of LV dysfunction to prevent systemic embolization is preferred [22-23]. A systematic review by de Gregorio found that early anticoagulation is beneficial in TTS in patients with or without thromboembolism [17]. This is also supported by an observational study by Santoro et al. that found that early heparin administration followed by warfarin for three months prevented stroke in TTS patients with and without LV thrombus [6].

## Conclusions

In conclusion, we have reviewed the most recent observational studies and clinical trials to summarize the management of TTS. The initial management should include optimal medical therapy with antiplatelet medications, statins, beta-blockers, and ACE/ARBs. Pressor medications are contraindicated in TTS with LVOTO. In TTS with thromboembolism, heparin with warfarin should be continued for at least three months.
